# A sympathetic gut connection drives the metabolic benefits of Roux-en-Y gastric bypass

**DOI:** 10.15698/cst2020.12.236

**Published:** 2020-11-24

**Authors:** Mohammed K. Hankir

**Affiliations:** 1 Department of Experimental Surgery, University Hospital Wuerzburg, Wuerzburg, 97080, Germany.

**Keywords:** Roux-en-Y gastric bypass, sleeve gastrectomy, resting metabolic rate, thermogenesis, splanchnic nerve, mesenteric white fat browning, weight loss, cannabinoid 1 receptor

## Abstract

Surgery is regarded by many as the go-to treatment option for severe obesity; yet how physically altering the gastrointestinal tract produces such striking results on body weight and overall metabolic health is poorly understood. In a recent issue of *Cell Reports* Ye *et al.* (2020) compare mouse models of Roux-en-Y gastric bypass (RYGB) and sleeve gastrectomy (SG), the two most commonly performed weight loss surgeries in the clinic today, to show that the former reconfiguring procedure selectively increases resting metabolic rate through splanchnic nerve-mediated browning of mesenteric white fat. More significantly, they demonstrate that this effect for RYGB is required for the maintained negative energy balance and improved glycemic control that it confers.

As the global incidence of obesity continues to rise, so too does the number of weight loss surgeries being performed [1]. Nevertheless, demand for these surgeries still far outweighs their supply [1], and current noninvasive alternatives remain only a fraction as effective [2]. For these reasons, research into how RYGB and SG in particular confer their pronounced weight-lowering effects and metabolic benefits to patients with severe obesity has grown in prominence. This has led to the development and refinement of RYGB and SG mouse models by various independent laboratories [3]. Where these models have largely succeeded in reproducing the weight loss trajectories observed for RYGB and SG proper [3], they have fallen short in reproducing their lasting suppression of food intake [4]. They do, however, successfully reproduce (perhaps even too well) the post-RYGB and SG increases in resting metabolic rate reported in clinical studies [4]. Consequently, when functional brown fat was rediscovered in adult humans a little over ten years ago now [5–7] and white fat browning (also known as britening or beiging) gained mainstream attention by the metabolic community, it soon followed that surgical scientitsts would ask how these thermogenic and glucoregulatory tissues are affected by RYGB and SG [8]. The results would be decidedly mixed, with some evidence of enhanced brown fat thermogenesis, white fat browning, or neither, for both procedures [8]. These inconsistencies aside, the jury was still very much out concerning the causal role of thermogenesis in mediating the outcomes of RYGB and SG on body weight and glycemic control.

Writing in *Cell Reports*, Ye *et al.* (2020) [9] directly compared resting metabolic rates in RYGB and SG mouse models. Where their approach differs from previous preclinical and clinical studies on the subject is how they opted to perform both direct and indirect calorimetry measurements [8]. From this, they could cleverly deduce that at a stage when both procedures induced similar weight loss, only RYGB-operated mice had higher total and anaerobic resting metabolic rates compared with sham-operated mice. Guided by these findings, the authors proceeded to analyse various fat depots for molecular markers of thermogenesis such as the inner mitochondrial membrane proton symporter uncoupling protein 1 (UCP1). Surprisingly, they found that *Ucp1* mRNA levels were lower in classical (interscapular) brown fat and subcutaneous (inguinal) white fat of RYGB-operated compared with sham-operated mice. In contrast, *Ucp1* mRNA levels were twice as high in visceral (mesenteric) white fat along with higher UCP1 protein levels. Again, analysis of this particular fat depot, which lines the outside of the gastrointestinal tract and as such is regarded as the true visceral fat depot, is what sets the study of Ye *et al.* [9] apart from the rest [8]. While almost entirely overlooked in the context of thermogenesis, mesenteric white fat has been shown to increase *Ucp1* mRNA levels by approximately 4-fold in rats chronically exposed to cold [10]. In line with the mesenteric white fat browning of RYGB-operated mice, their uptake of a radioactive glucose analogue in the small intestinal region was 50% higher compared with body weight-matched sham-operated mice, although it should be noted that this does not necessarily reflect thermogenesis *per se* [11] or could simply be attributable to heightened metabolic activity of jejunal enterocytes [12].

Next, because the sympathetic nervous system (SNS) is a major driver of thermogenesis [13–15], Ye *et al.* (2020) [9] assessed molecular markers of sympathetic tone in jejunal and mesenteric white fat samples such as the rate-limiting enzyme in noradrenaline production tyrosine hydroxylase (TH). Curiously, this revealed higher TH protein levels in human and mouse jejunal mucosa after RYGB where sympathetic nerve terminals do not normally reach. There was also higher TH protein levels in mesenteric white fat of RYGB-operated compared with sham-operated mice, providing first evidence of enhanced sympathetic tone in this region. To more directly prove this, the authors performed intricate electrophysiological recordings of the greater splanchnic nerve, a mixed sympathetic nerve with cholinergic efferents that originate in the lateral horn of the thoracic spinal cord and whose axons pass straight through the sympathetic trunk to synapse at the celiac ganglion with noardrenergic efferents that innervate the small intestine. It was found that RYGB-operated mice had higher neuronal activity compared with sham-operated mice both before and during weight loss. Additionally, by transecting the greater splanchnic nerve distal to the recording electrode (just proximal to the celiac ganglion), the authors could silence afferent fibre activity and uncover higher efferent fibre activity for RYGB-operated mice. Notably, the electrical activity of sympathetic fibres innervating interscapular brown fat was similar between the two surgical groups, suggesting that reconfiguring the gastrointestinal tract causes region-specific changes in sympathetic tone.

From these results, Ye *et al.* (2020) [9] were now ideally placed to ask whether intact sympathetic innervation of the small intestine is required for the weight-lowering effects and metabolic benefits of RYGB. To do so, they selectively transected the lesser splanchnic nerve and removed the celiac ganglia all the while carefully preserving sympathetic innervation of the kidney and adrenal glands. Remarkably, this resulted in weight regain in RYGB-operated mice and abolishment of their higher total and aerobic resting metabolic rates as well as mesenteric white fat browning. Further, the improved insulin sensitivity of RYGB-operated mice was lost although their improved glucose tolerance was largely preserved: unexpected as the avid glucose uptake of thermogenic adipocytes when sympathetic tone is high should also be lost upon sympathetic denervation [16] with corresponding effects on glycemic control.

Finally, to identify a mechanism for increased splanchnic nerve activity after RYGB, Ye *et al.* (2020) [9] systematically considered various possibilities. Circulating gut hormones such as glucagon-like peptide 1 (GLP-1) and peptide tyrosine tyrosine (PYY) as well as bile acids were excluded since they were equally increased by RYGB and SG, although specific bile acid species could be differentially regulated by the two procedures. Indeed, the bile acid receptor Takeda G-protein coupled receptor 5 (TGR5) has previously been shown to be required for weight loss, increased resting metabolic rate, and interscapular brown fat themogenesis for SG [17] but not for RYGB [18] in diet-induced obese mice. The authors then narrowed their search down to the endocannabinoid system because of its established role in regulating resting metabolic rate and energy balance. By measuring cannabinoid 1 (CB1) receptor protein levels in jejunal whole-wall lysates, they found what appeared to be a complete disappearance for RYGB-operated mice unlike for SG-operated mice. Further, the effects of RYGB on body weight and mesenteric white fat browning were partially reversed with the endogenous CB1 receptor agonist anandamide, although in principle this would have been occluded by the absence of jejunal CB1 receptors. Providing theraputic value to their findings, they could demonstrate that chronic oral administration of the synthetic CB1 receptor inverse agonist rimonabant to diet-induced obese mice mimicked some of the key features of RYGB such as higher splanchnic nerve efferent activity, mesenteric white fat browning, and weight loss. This latter set of pharmacological experiments also provides important proof-of-principle that counteracting intestinal CB1 receptor signaling is sufficient to enhance local sympathetic tone.

The findings of Ye *et al.* (2020) [9] offer an unprecedented level of mechanistic insight into how RYGB produces such striking results on body weight and overall metabolic health, but several key questions remain. For example, how rerouting ingested food pasage from the (smaller) stomach away from the duodenum and directly to the jejunum causes downregulation of jejunal CB1 receptors after RYGB was not established, nor was it possible for the authors to measure jejunal endocannabinoid levels. Interestingly, RYGB has previously been shown to robustly decrease endocannabinoid levels (anandamide and 2-arachidonoylglycerol) in the liver and skeletal muscle of diet-induced obese rats independently of weight loss [19]. This suggests that RYGB decreases CB1 receptor (and/or CB2 receptor) signaling in various peripheral tissues, which may confer distinct metabolic benefits. Additionally, the cell types that contribute to downregulation of jejunal CB1 receptors after RYGB and the precise neural circuit that this would then recruit to increase splanchnic nerve outflow is unclear (**Figure 1**). Enteroendocrine cells in the upper small intestine and vagal afferent neurons express CB1 receptors [20, 21], but their selective deletion in both sensory cell types does not promote weight loss [22, 23] and RYGB retains its metabolic benefits in mice lacking GLP-1 and Y2 receptors [24] or an intact vagus nerve [25]. We are then left with intestinal immune cells residing in the lamina propria. Despite the lack of overt changes in the jejunal immune cell landscape of RYGB-operated mice reported by Ye *et al.* (2020) [9], the possibility still exists that CB1 receptors are downregulated in distinct innate and/or adaptive immune cell types [26], which in turn could regulate the excitability of splanchnic afferent neurons innervating the jejunal mucosa through an unknown secreted factor (**Figure 1**). Indeed, Ye *et al.* [9] found that denervation of the greater splanchnic nerve in itself led to weight loss in diet-induced obese mice due to reduced food intake, attesting that gut-derived signals are relayed via splanchnic afferent neurons to the central nervous system to regulate energy balance. Another question that remains is to what extent the well established shifts in the intestinal microbiota following RYGB contribute to mesenteric white fat browing. Ye *et al.* (2020) [9] found a splanchnic nerve-mediated decrease in cecal *Bacteroidetes* in RYGB-operated mice, which is in line with how this bacterial phyla negatively associate with thermogenic markers in subcutaneous white fat of obese patients [27]. It is also possible that splanchnic efferents regulate microbiota residing in mesenteric white fat itself [28] following RYGB, to influence various cellular processes including thermogenesis. Lastly, since RYGB increases anaerobic resting metabolic rate, a non-UCP1-dependent form of thermogenesis in mesenteric adipocytes may become operational, as UCP1 activation results in oxygen consumption [29]. This could potentially be fulfilled by anaerobic glycosis, which occurs when glucose metabolism predominates in cells and glycolytic rates are high, and can have a sizeable impact on organismal thermogenesis [30]. Going forward, the groundbreaking study of Ye *et al.* (2020) [9] confirms how garnering better insight into the mechanistic underpinnings of weight loss surgeries can delineate new pathways that regulate energy balance to potentially guide the development of alternative, non-invasive treatments for obesity and its comorbidities.

**Figure 1:**
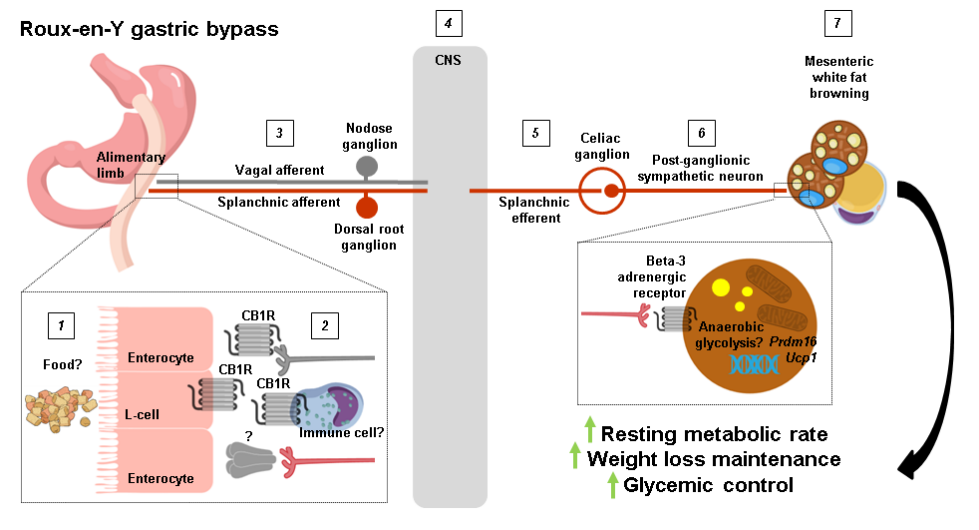
FIGURE 1: A proposed pathway for how RYGB increases resting metabolic rate. **1** The rerouting of ingested food caused by RYGB may **2** downregulate jejunal CB1 receptors in L-cells, peripheral vagal afferent endings, and/or immune cells. This would then result in **3** the increased excitability of vagal afferent and splanchnic afferent nerve fibers which propagate their signals to **4** the central nervous system where they are processed. Splanchnic efferent neurons are in turn **5** stimulated to **6** region-specifically increase sympathetic tone and **7** induce browning of mesenteric white fat. The ultimate outcome is an increase in resting metabolic rate which drives weight loss maintenance and improvements in glycemic control.

## Abbreviatons:

CB1: – cannabinoid 1;
RYGB: – Roux-en-Y gastric bypass;
SG: – sleeve gastrectomy;
TH: – tyrosine hydroxylase;
UCP1: – uncoupling protein 1.
